# The Role of Periprostatic Adipose Tissue on Prostate Function in Vascular-Related Disorders

**DOI:** 10.3389/fphar.2021.626155

**Published:** 2021-02-12

**Authors:** Gabriela Reolon Passos, Ana Carolina Ghezzi, Edson Antunes, Mariana Gonçalves de Oliveira, Fabiola Zakia Mónica

**Affiliations:** Department of Pharmacology, Faculty of Medical Sciences, University of Campinas (UNICAMP), Campinas, Brazil

**Keywords:** prostate, periprostatic adipose tissue, obesity, adipokines, benign prostate hyperplasia

## Abstract

The lower urinary tract symptoms (LUTS) secondary to benign prostatic hyperplasia (BPH) are highly prevalent worldwide. Clinical and experimental data suggest that the incidence of LUTS-BPH is higher in patients with vascular-related disorders such as in pelvic ischemia, obesity and diabetes as well as in the ageing population. Obesity is an important risk factor that predisposes to glucose intolerance, insulin resistance, dyslipidemia, type 2 diabetes mellitus and cardiovascular disorders. Prospective studies showed that obese men are more likely to develop LUTS-BPH than non-obese men. Yet, men with greater waist circumferences were also at a greater risk of increased prostate volume and prostate-specific antigen than men with lower waist circumference. BPH is characterized by an enlarged prostate and increased smooth muscle tone, thus causing urinary symptoms. Data from experimental studies showed a significant increase in prostate and epididymal adipose tissue weight of obese mice when compared with lean mice. Adipose tissues that are in direct contact with specific organs have gained attention due to their potential paracrine role. The prostate gland is surrounded by periprostatic adipose tissue (PPAT), which is believed to play a paracrine role by releasing growth factors, pro-inflammatory, pro-oxidant, contractile and anti-contractile substances that interfere in prostate reactivity and growth. Therefore, this review is divided into two main parts, one focusing on the role of adipokines in the context of obesity that can lead to LUTS/BPH and the second part focusing on the mediators released from PPAT and the possible pathways that may interfere in the prostate microenvironment.

## Introduction

The prostate gland is a reproductive organ whose main function is to secrete an alkaline fluid that, along with sperm cells from the testicles and fluids from other glands, makes up the semen (Verze et al., 2016). The human prostate is divided into three zones, according to John McNeal (McNeal 1984), namely the peripheral zone (contains the majority of glandular tissue), the central zone (surrounds the ejaculatory ducts) and the transition zone (surrounds the urethra just beneath the bladder). The enlargement of the transition zone is considered the main cause of benign prostatic hyperplasia (BPH) in ageing men and is one of the main causes of lower urinary tract symptoms (LUTS) (Roehrborn 2008; D’Ancona et al., 2019).

**FIGURE 1:**
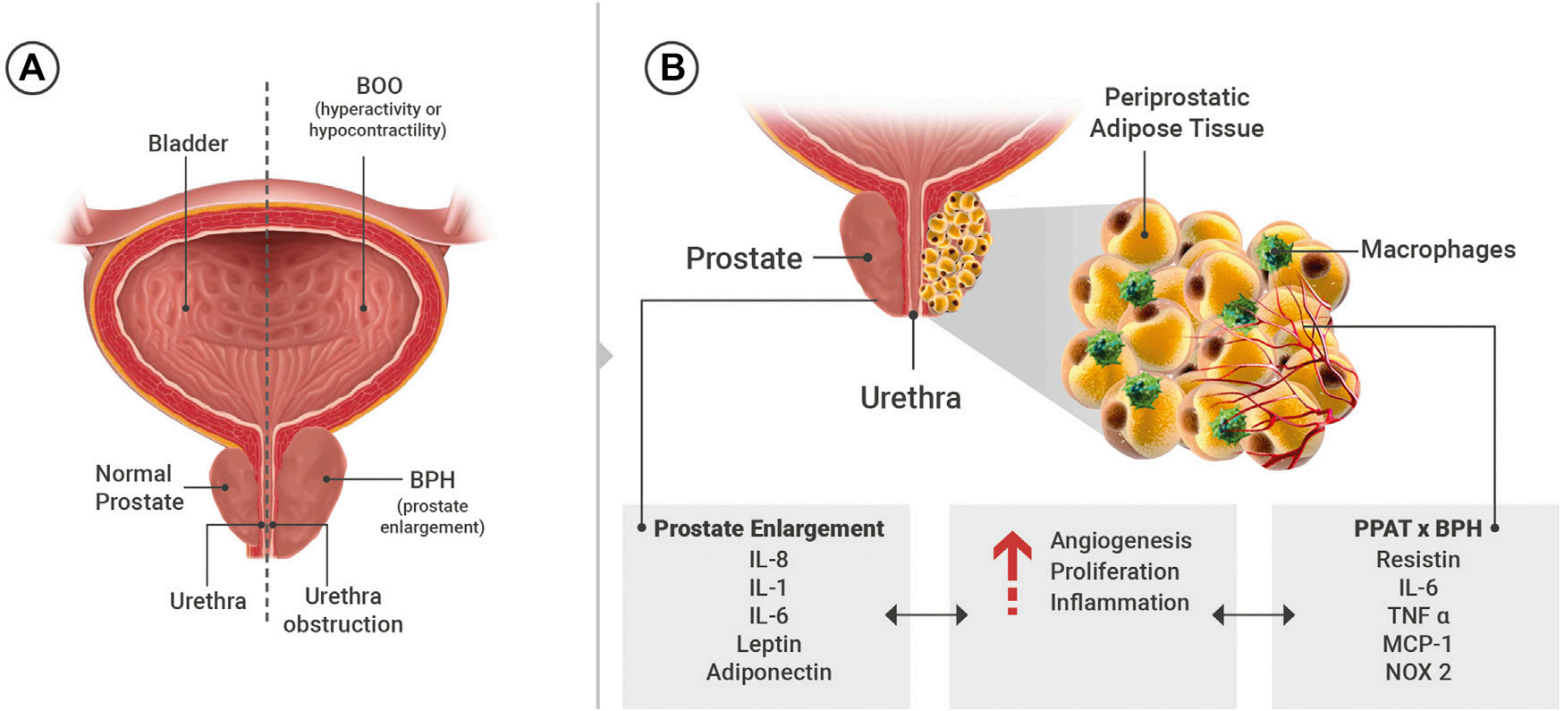
**(A)** General scheme showing the organs of the lower urinary tract. **(B)** Substances present in the systemic circulation and/or released from periprostatic adipose tissue (PPAT) that may interfere in the prostate microenvironment such as angiogenesis, proliferation and inflammation. IL: interleukins, TNF: tumor necrosis factor-α, NADPH oxidase 2 (NOX 2), MCP-1: monocyte chemoattractant protein-1.

LUTS refer to a group of clinical symptoms that involve the bladder, urinary sphincter, urethra and, in men, prostate. LUTS are divided into three main groups that are storage, voiding and post micturition symptoms. *Storage symptoms* are observed during the storage phase of the bladder and can be classified into i) general storage symptoms, ii) sensory symptoms and iii) incontinence symptoms. *Voiding symptoms* are LUTS experienced during micturition (hesitancy, episodic inability to void, straining to void, slow stream, intermittency, terminal dribbling, spraying, dysuria, hematuria, pneumaturia and urinary retention) (Figure 1A). Post-micturition symptoms are experienced after voiding ceases and are characterized by a feeling of incomplete bladder emptying, a need to immediately re-void, post-void incontinence and post-micturition urgency (D'Ancona et al., 2019). The prevalence of LUTS is over 60% in men and women aged > 40 years (Irwin et al., 2006; Coyne et al., 2009) and are also associated with various modifiable risk factors such as obesity, diabetes, and metabolic syndrome (Gacci et al., 2015; Chughtai et al., 2016). LUTS interfere in the quality of life, sexual quality, social functioning and productivity at work. The therapeutic management of LUTS secondary to BPH is aimed at relaxing the bladder (antimuscarinics, beta-3 adrenoceptors agonist) and/or prostatic smooth muscle (alpha-1 antagonists, phosphodiesterase type 5 inhibitors) and to inhibit prostate proliferation (5-alpha reductase inhibitors) (Morelli et al., 2011; Mónica and De Nucci, 2019).

The overproduction of testicular androgens is considered a key step in the development of BPH. The enzyme 5-alpha reductase type 2 converts testosterone into dihydrotestosterone (DHT), the main intraprostatic androgen. The imbalance between cell proliferation and cell death is a proposed mechanism for BPH progression (Carson and Rittmaster, 2003). DHT, which is more potent than testosterone, translocates to the nucleus and favors the transcription of several growth factors such as keratinocyte growth factor, epidermal growth factor and insulin-like growth factor (Chughtai et al., 2016).

Clinical and experimental data show a greater prevalence of LUTS in patients who present metabolic disorders that predispose to various diseases including obesity and BPH. The prostate gland is surrounded by the periprostatic adipose tissue (PPAT), which is believed to play a paracrine role by releasing anti-and pro-inflammatory substances, growth factors, contractile and anti-contractile substances. Therefore, this review is divided into two main parts, one highlighting the role of adipokines in the context of obesity that can lead to LUTS/BPH and the second part the role of substances released from PPAT that may facilitate the development of prostate growth.

## Obesity as Risk Factor

Obesity is an epidemic health problem worldwide. A 20% increase in body weight increases significantly the risk of developing insulin resistance, dyslipidemia, type 2 diabetes mellitus and other cardiovascular diseases (World Health Organization, 2002). A strong association between heritability and obesity (Friedman, 2016; Chiurazzi et al., 2020; Lan et al., 2020) and low-grade systemic inflammation (Xu et al., 2003; Hotamisligil 2006) also exist. Adipokines which are released from adipose tissue, may also engage cells of the immune system that can also contribute to the chronic inflammatory state seen in obesity (Bouloumié et al., 2005).

Mice and rats fed with a high fat diet showed marked increases in the body and prostate weights (Silva et al., 2015; Calmasini et al., 2018), along with larger number of cells and blood vessels in the ventral prostate when compared to the prostates from lean animals (Silva, et al., 2015). *In vitro* studies reported a hypercontractile state of the prostate smooth muscle from obese animals as characterized by greater contraction induced by transmural stimulation or by direct activation of alpha-1-adrenoceptors (Silva, et al., 2015; Calmasini, et al., 2018). Another study has identified increased levels of phosphorylated-ERK (Extracellular Signal-Regulated Kinases) in the prostate from hyperinsulinemic rats fed with a high fat diet, suggesting an involvement of the MERK/ERK (Mitogen-activated protein kinases/Extracellular signal-regulated kinases) pathway in the prostate growth (Vikram et al., 2010).

The inflammatory microenvironment favors the proliferation of epithelial and stromal cells in BPH pathogenesis. Meng et al., 2020, included 77 patients (±69 years) with BPH who underwent transurethral prostatic resection and no report of LUTS complaint. In all biopsy samples, inflammatory infiltrates were identified. The predominant cell type was T lymphocytes, which were identified in 96% of samples, followed by B lymphocytes (77%) and macrophages (52.6%). Individuals with a waist circumference more than 100 cm had a higher incidence of moderate/severe LUTS, evaluated by an International Prostate Symptoms Score (IPSS) greater than eight for the initiation of the treatment of BPH, erectile and ejaculatory dysfunction, as well as a greater propensity to increased prostate volume (Penson et al., 2011). In Sweden, a survey with 27,858 men showed that participants exhibiting the highest abdominal obesity ratio were 22% more likely to suffer moderate to severe LUTS. Furthermore, for every 5-unit increase in BMI the risk of developing severe LUTS secondary to BPH increased by 13% as evaluated by IPSS (Laven et al., 2008).

## The Adipose Tissue

Three types of adipocytes are identified, commonly classified based on their morphology and function. The White Adipose Tissue (WAT), composed by a unilocular lipid droplet that occupies > 95% of the adipocyte, is composed of non-thermogenic and energy-storing type of fat cells. Brown Adipose Tissue (BAT), composed of multilocular lipid droplets dispersed in a mitochondrial rich cytoplasm, acts in thermogenesis, which is mediated by uncoupling protein 1 (UCP-1) (Jeremic et al., 2017; Lu et al., 2020). Beige adipose tissue has now been identified as a cluster in WAT, with multilocular/unilocular morphology and able to both store and spend energy (Cheng et al., 2019; Mao et al., 2020).

The stimulation of beta-adrenoceptors (ARs) in the WAT and BAT is crucial for the activation of thermogenic pathways. The beta- ARs are the main adrenergic receptors involved in pathways related to WAT browning and BAT thermogenesis, although the subtypes involved differ between species. For example, in rodents, beta3-AR agonists increase lipolysis, fat oxidation and energy expenditure (Galitzky et al., 1993; Uehling et al., 2006; Michel and Korstanje, 2016; Hao et al., 2019), thus leading to the belief that this class of substances would be effective in the treatment of diabetes and obesity. However, in humans, lipolysis occurs mainly via beta-1AR (Rosenbaum et al., 1993; Barbe et al., 1996; Michel and Korstanje, 2016). The differences between species (rodents vs human), the lack of selective tools (antibodies, agonists and antagonists) to differentiate beta3-AR over beta1-/beta2-ARs and the absence of clinical data make beta3-AR agonists ineffective in the treatment of obesity and type 2 diabetes. On the other hand, in the human bladder, beta3-AR is the main subtype that induces smooth muscle relaxation (Nomiya and Yamaguchi, 2003). To date, mirabegron, a beta3-AR agonist is used to treating patients with overactive bladder.

## Adipose Tissue in Obesity

Under conditions of over-nutrition, an expansion of WAT, a remodeling of extracellular matrix components and blood vasculature, along with an enhanced secretion of pro-inflammatory mediators all contribute to local and systemic inflammation (Vgontzas et al., 2000). Adipose tissue is mainly formed by adipocytes, but other cell types also contribute to its growth and function such as pre-adipocytes, lymphocytes, macrophages, fibroblasts and vascular cells (Tilg and Moschen, 2006; Ouchi et al., 2011). WAT exerts endocrine and paracrine functions by releasing peptides, non-peptides and cytokines called adipokines. Adipokines have highly diverse chemical structures and physiological functions. For example, some adipokines are involved in inflammation such as the cytokines; Tumor Necrosis Factor - alpha (TNF-α), interleukin-1 [IL-1], IL-6, monocyte chemoattractant protein-1 (MCP-1) while others regulate feeding behaviour through the central nervous system (leptin), sensitize insulin (adiponectin) or have anti-inflammatory properties (adiponectin, secreted frizzled-related proteins) (Ouchi et al., 2011). In humans, leptin, adiponectin, resistin and visfatin are key adipokines that work as hormones to interfere in energy haemostasis and to regulate neuroendocrine function. Adipokines that have cytokine properties interfere mainly in immunological and inflammatory processes, both locally and systemically (La Cava and Matarese, 2004; Kusminski et al., 2007; Tilg and Moschen, 2006; Carr and Rodeo, 2019; Smith et al., 2020).

Leptin, along with adiponectin, are the main adipokines released from the adipose tissue, although they are not all exclusively derived from this organ. In mammals, the action of leptin in the central nervous system reduces food intake and increases energy expenditure, in addition to regulating neuroendocrine function and glucose and fat metabolism (Friedman, 1997). Animals that are leptin-deficient present defects in the neuroendocrine axis, are infertile or subfertile and present greater levels of corticosterone. Male ob/ob mice treated with leptin for 15 and 75 days were used to evaluate energy expenditure thorough food intake and body composition. The body mass of ob/ob mice decreased around 30% in 15 days of leptin treatment and the food consumption of the animals was reduced by about 95%. Leptin-induced reduction of food intake ceased during treatment with leptin over 75 days, when the lipid reserves of the mice were depleted and energy expenditure became similar to that in control mice (Rafael and Herling 2000). In mice, the activation of sympathetic efferent nerves to adipose tissue was seen to be involved in the leptin-induced lipolysis in WAT (Zeng et al., 2015). While in mice a defect in leptin axis promotes hyperphagia and a decrease in energy expenditure, in humans, leptin acts mainly on appetite (Friedman, 2016). However, obese individuals have elevated plasma leptin in comparison with non-obese individuals. The hyperleptinemia found in obese individuals is attributed to alterations in the leptin receptor or a deficiency in its transport, a phenomenon called leptin resistance (Considine et al., 1996). The beneficial effects of the treatment with leptin in obese patients are controversial. Four weeks treatment of exogenous leptin in both eutrophic and obese individuals reduced body weight (Friedman and Hallas, 1998), however, this effect was only observed in those individuals who did not present hyperleptinemia, as the administration of leptin in obese patients resistant to leptin did not produce a significant weight loss (Maffei et al., 1995; Lee et al., 2002). Leptin receptors are also found in endothelial cells and may induce reactive oxidative stress, which can aggravate cardiometabolic problems (Bouloumie et al., 1999; Yamagishi et al., 2001).

Adiponectin, in turn, has a cardioprotective role (Matsubara et al., 2002). He et al., 2016 showed that TNF-α impairs adiponectin multimerization and, consequently, decreases adiponectin secretion both *in vitro* and *in vivo*. Adiponectin overexpression in leptin-deficient *ob/ob* mice induces the redirection of adipose tissue deposits from the visceral region to the subcutaneous region, promoting an improvement in blood glucose, systemic insulin sensitivity, dyslipidemia and inflammation (Kim et al., 2007). The cardioprotective effect promoted by adiponectin has been partially explained by its anti-inflammatory role. In co-culture of human skeletal muscle cells with human adipocytes adiponectin negatively modulates the release of IL-6, IL-8 TNF-α and MCP-1 in the adipose tissue, which are linked to insulin resistance (Dietze-Schroeder et al., 2005). Adiponectin also releases IL-10 secretion, an anti-inflammatory cytokine that plays an important role in the polarization of macrophages to the M2 profile (Kumada et al., 2004).

## Role of Adipokines in Prostate Growth

Inflammatory cells release cytokines and growth factors that may contribute to prostate growth (Steiner et al., 2003; Delongchamps et al., 2008). For instance, in primary culture of prostatic epithelial cells, IL-8 increased the expression of the fibroblast growth factor (FGF) and its blockade reduced the production of FGF. In specimens from patients with BPH, the expression of IL-8 was greater in epithelial cells than in normal prostate (Giri and Ittmann, 2001). The levels of IL-8 and its receptors were up-regulated in BPH tissues when compared to normal tissues. In BPH-1 cells, the IL-8 axis was increased in comparison with normal epithelial cells and the deletion of its receptor, CXR7 inhibited the growth (Smith et al., 2020). A study with normal, BPH and PCa prostate tissue found greater expression of IL-1 and IL-6 in specimens from BPH and PC samples in the epithelial and stromal compartments, thus suggesting that these cytokines may play a role in epithelial hyperplasia (Mechergui et al., 2009).

Prostates removed from leptin-deficient ob/ob male mice that received testosterone (3 mg/kg for 14 days) to induce BPH, showed a smaller proportion of glandular lumen and reduced collagen deposition in comparison to prostates from control and ob/ob mice. Because extracellular deposition and glandular hyperplasia are typical patterns of BPH, these results suggest that leptin deficiency attenuated morphological changes and collagen deposition. Yet, prostates from ob/ob + testosterone exhibited greater levels of E-cadherin and lower levels of vimentin in comparison to the testosterone control group. These results suggested that leptin may function as a facilitator of the epithelial-mesenchymal transition (EMT) to favor BPH progression (Zhang et al., 2020).

The receptors for adiponectin; AdipoR1 and AdipoR2 are expressed in normal epithelial and stromal cells lines (RPWE and WPMY1), as well as in the stroma and epithelium in specimens from BPH. *In vitro* addition of adiponectin in RPWE and WPMY1 cells arrested cells in G0 phase and induced apoptosis by increasing the expression of caspase-3. In obese mice fed with a high-fat diet, treatment with recombinant adiponectin for 14 days protected the prostate from histologic BPH, thus showing that adiponectin reduced prostate growth (Fu et al., 2017).

## Relationship Between Periprostatic Adipose Tissue and Prostate Growth

Adipose tissues that are in direct contact with specific organs have gained attention due to their potential paracrine role. Among them are perivascular adipose tissue (PVAT), which comprises 3% of all adipose tissue mass and surrounds the vessels (Szasz et al., 2013), and PPAT, which surrounds the prostate and the prostatic urethra. The anatomic distribution of PPAT was evaluated in radical prostatectomy specimens. The presence of PPAT was along 48% of the prostate surface, of that 57-59% present PPAT on the right and lateral surface and 44% and 36% along the anterior and posterior region, respectively (Hong et al., 2003). PPAT can have similar characteristics to PVAT by releasing pro-inflammatory cytokines (resistin, IL-6, TNF-α, MCP-1, chemerin, progranulin) (Blüher 2013; Alexandre et al., 2018), growth factors (progranulin and chemerin) (Klöting et al., 2010), contractile (angiotensin II, superoxide generation) (Lu et al., 2010) and anti-contractile (leptin, adiponectin, H_2_O_2_, ADRF and NO) (Victorio et al., 2016; Araujo et al., 2018) substances (Figure 1B).

Most studies that have evaluated the role of substances released from PPAT on prostate focused on cancer. Although that there is no evidence to suggest that men with LUTS due to BPH are at an increased risk of prostate cancer, in this section we focused only on experimental studies that evaluated the possible interplay between PPAT and BPH due to word limit. More information about PPAT in cancer can be seen in the studies conducted by Finley et al., 2009 and Ribeiro et al., 2012 (Table 1).

**TABLE 1:** Major pre-clinical and clinical findings linking the role of PPAT, adipokines and lower urinary tract dysfunction.

Specie	Protocol design	Key results	Reference
Human	Specimens from patients with BPH	The inflammation associated with BPH is characterized by greater number of T-cells and greater expression of the cytokine IL-1.	Steiner et al. (2003)
Human	PPAT anatomic distribution	Presence of PPAT was along the 48% of prostate surface, of that 57-59% represent the PPAT on the right and lateral surface, 44% and 36% along the anterior and posterior region, respectively.	Hong et al. (2003)
Human	Normal, BPH and PCa prostate tissue	Greater expression of IL-1 and IL-6 in specimens from BPH and PC sample in the epithelial and stromal compartments in comparison with normal prostate.	Mechergui et al. (2009)
Human	PPAT explants from patients with prostate cancer	Higher secretion of IL-6 from PPAT, which was correlated with higher tumor grade. Increased phosphorilation in Jak/Stat, Akt/mTOR and NFκB pathways identified as IL-6 downstream.	Finley et al. (2009)
Human	Cultured PPAT and stromal vascular fraction (SVF) from BPH patients	In PPAT explants increased proteolitic activities and up-regulation of MMP2 and MMP9 in comparison with SVF	Ribeiro et al. 2012
Human	Prostate cancer cells lines	PPAT supernatants-derived factors increased migration of both PC3 and LNCaP cells, while PPAT had a strong proliferative effect on PC3.	Ribeiro et al. (2012)
Human and mice	RPWE and WPMY1 and in specimens from patients with BPH.	Addition of adiponectin in RPWE and WPMY1 cells arrested cells in G_0_ phase and induced apoptosis. Long-term treatment with adiponectin protected the prostate from histologic BPH in obese mice	Fu et al. (2017)
Mouse	Urethral smooth muscle from high fat diet-induced obesity	Obese mice exhibited urethral hypercontractility and reduced NO-induced relaxation response along with increased PPAT size, higher ROS generation besides NOX2 and TNFα overexpression.	Alexandre et al. (2018)
Human	Conditioned media from PPAT obtained from patients with prostate cancer and BPH	The integrin family cells surface interaction, homeostasis pathway and TRAIL signaling pathway were the most enriched pathway for CM-T3, CM-T2 and CM-BPH, respectively.	Sacca et al. (2019)
Human	BPH patients and BPH-1 cells	The levels of IL-8 and its receptors were up-regulated in BPH tissues when compared to normal tissues. In BPH-1 cells the IL-8 axis was increased in comparison with normal epithelial cells and the deletion of its receptor, CXR7 inhibited the growth by 50%	Smith et al. (2020)
Human and mice	Prostates from ob/ob mice that received testosterone and BPH-1 cells	Leptin deficiency attenuated morphological changes and collagen deposition. In BPH-1 cells treated with leptin a decrease and increase protein expression of E-cadherin and vimentin, respectively, were observed.	Zhang et al. (2020)

In obese mice, the area of PPAT and the gene expression for NADPH oxidase 2 (NOX 2) and TNF-α were all higher than in the lean mice. Although this study did not evaluate the impact of PPAT on the prostate, it strongly suggests that PPAT may release pro-inflammatory and pro-oxidant substances that could interfere in prostate contractility (Alexandre et al., 2018).

The secretome derived from conditioned media from PPAT (PPAT-CM) obtained from patients with prostate cancer and BPH were assessed. The authors observed that the amount of protein in PPAT-CM derived from patients with prostate cancer stage 3 (PCa-T3) were significantly higher, relative to CM-PCaT2 and CM-BPH. Proteins related to lipid transport and adipogenesis were detected in all three CM. Regarding pathways related to biological functions, the integrin family cell surface interaction, homeostatic pathway and TNF-related apoptosis-inducing ligand/Apo-2L (TRAIL) signaling pathway were the most enriched pathways for CM-T3, CM-T2 and CM-BPH, respectively, which means that in the case of cancer cells, overexpression of these biological pathways is related to cell adhesion, migration and invasiveness (Sacca et al., 2019). In this study it is not possible to know the source of these proteins, as in PPAT-CM there are different cell types. According to the study above, TRAIL was the most enriched biological pathway observed in CM-BPH. In a cell line from the epithelium of a hyperplasic prostate, BPH-1 cells, which express low abundance of the protein DJ-1 (a protein involved in transcriptional regulation, antioxidative stress reaction and chaperone) in comparison with cancer cells (LNCaP, Du145, PC-3), the addition of increasing concentrations of TRAIL correlated with an increased the expression of DJ-1. Yet the accumulation of TRAIL increased DJ-1 in treated BPH-1 cells, occurred in a time-dependent manner, reaching its peak after 25 min. These results suggest that DJ-1 plays a role in the control of apoptosis (Hod, 2004).

## Final Remarks: Potential Therapies Targeting Adipose Tissue

Cardiometabolic risk factors affect the function of different adipose tissue deposits. Ageing, sex steroid hormones, metabolic syndrome, cardiovascular diseases, inflammation and obesity are modifiable and non-modifiable risk factors that contribute to the pathogenesis of BPH. Obesity is a component of metabolic syndrome and both are associated with systemic and local inflammation. PPAT, which surrounds part of the prostate surface, has been implicated in the release of paracrine factors that may lead to the development of BPH. The therapeutic aim in BPH is to improve symptoms and lower the risk of progression to improve patient quality of life. Different pharmacological classes of drugs are used in LUTS-BPH including those to reduce outlet obstruction and to treat bladder overactivity. The role of PPAT dysfunction as a trigger of BPH, especially in cases of obesity, is a new area of investigation and more studies are needed to find key mediators directly involved in the pathogenesis of prostatic hyperplasia.
